# Effects of cyclin-dependent kinase inhibitor Purvalanol B application on protein expression and developmental progression in intra-erythrocytic *Plasmodium falciparum* parasites

**DOI:** 10.1186/s12936-015-0655-x

**Published:** 2015-04-08

**Authors:** Kristen M Bullard, Carolyn Broccardo, Susan M Keenan

**Affiliations:** School of Biological Sciences, University of Northern Colorado, Greeley, CO 80639 USA; Proteomics and Metabolomics Facility, Colorado State University, Fort ColliHns, CO 80523 USA

**Keywords:** *Plasmodium falciparum*, Malaria, Purvalanol B, Proteome, Proteomics

## Abstract

**Background:**

The 2013 Malaria World Report indicated that in 2012 there were approximately 207 million cases of malaria, which resulted in an estimated 627,000 malaria-related deaths. Due to the alarming resistance of these parasites to traditional anti-malarial treatments there is a pressing need to not only identify new anti-malarial compounds, but also to characterize the effect of compounds known to have an effect on the parasite life cycle. This study reports on effects of kinase inhibitor Purvalanol B administration on the growth and protein expression of *Plasmodium falciparum* late-stage trophozoites.

**Methods:**

A SYBR® Green I parasite growth assay was used to measure the IC_50_ of Purvalanol B with *P. falciparum* (strain W2). Purvalanol B or DMSO control were applied to synchronized parasites 36 hours post invasion and parasites were incubated for 12 hours. Giemsa-stained blood smears were used to determine the effect of Purvalanol B on parasite growth, global quantitative proteomic analysis was used to examine differences in protein expression between Purvalanol B-treated and control parasites and results were confirmed by qPCR.

**Results:**

There were no differences in parasitaemia between inhibitor-treated and control parasites. However, the ability of Purvalanol B-treated parasites to form schizonts was significantly reduced. Proteomic analysis detected 76 human proteins and 518 *P. falciparum* proteins (63 in control cultures only, 56 proteins in Purvalanol B cultures only, and 399 proteins in both cultures). Quantitative analysis of protein extracts revealed eight proteins that were up-regulated in the inhibitor-treated cultures, including several components of the parasite’s proteasome complex and thioredoxin reductase. Two proteins appeared to be down-regulated, including a helicase and an RNA-binding protein.

**Conclusion:**

Purvalanol B application decreases the ability of late-stage *P. falciparum* trophozoites to form multinucleated schizonts and up-regulates proteasome subunits and proteins that contribute to redox homeostasis, which may indicate an increase in oxidative stress as a result of inhibitor application. While the efficacy of Purvalanol B is relatively low for use as an anti-malarial therapy, quantitative proteomic analysis may serve as a method of examining the action of drugs on the parasite and indicate the likelihood of future resistance development.

**Electronic supplementary material:**

The online version of this article (doi:10.1186/s12936-015-0655-x) contains supplementary material, which is available to authorized users.

## Background

In 2013 the World Malaria Report issued by the World Health Organization estimated that 3.4 billion people were at risk of malaria infection and that this arthropod-borne disease took the lives of approximately 627,000 people in 2012 [1]. Malaria caused by *Plasmodium falciparum* remains the most devastating form of malaria and is a significant killer of children under five years of age in sub-Saharan Africa [2]. Given the number of people at risk of malaria infection and the increasing resistance of this parasite to traditional anti-malarials, such as artemisinin and its derivatives [3-5], there is a pressing need to identify new compounds with anti-malarial activity and to keep new drugs in the drug discovery pipeline. However, given the propensity of this parasite to develop drug resistance (as reviewed in [6]) it is also necessary to characterize the effects of promising new drugs on the parasite in order to help pinpoint the action of the drug and aid combinatorial and synergistic drug formulation strategies. Further, characterizing the effects of novels drugs on *P. falciparum* would help elucidate the timeline and mechanisms of resistance development. This study characterizes the effects of cyclin dependent kinase inhibitor Purvalanol B on intra-erythrocytic growth of *P. falciparum* using a quantitative proteomics approach.

In recent years, advances in quantitative proteomics have made possible the rapid identification of individual proteins in complex protein mixtures with much higher resolution than was previously possible. The comparison of global protein expression patterns between *P. falciparum* parasites in different life cycle stages [7-9], the identification of drug targets and resistance development [10,11], and the characterization of protein expression differences resulting from application of canonical anti-malarials, such as tetracycline, pyrimethamine, doxycycline, chloroquine, and artemisinin, have been described [8,12,13]. A quantitative proteomics approach may also be useful for describing the effect of new anti-malarials on *P. falciparum*. In 2005, a proteomics study of the then new combinatorial inhibitor CoArtem®, a formulation including artemether and lumefantrine, was able to show that the two drugs had opposite effects on glycolytic enzymes but similar effects on proteins expressed as a result of a stress stimuli, which elucidated the mechanism of action of the two active components of this drug therapy [14]. Comparing protein expression profiles from drug-challenged and non-treated parasites may shed light on the mechanism of action of novel anti-malarial drugs and may indicate whether certain drugs are more likely to result in parasite resistance.

To date, changes in the *P. falciparum* proteome in response to drug challenge with well-characterized anti-malarial drugs, such as chloroquine and artesunate, have been documented. However, there are relatively few studies using quantitative proteomics techniques to describe the effects of potential anti-malarial compounds. The small molecule, Purvalanol B was first described as a 2,6,9-trisubstituted purine and ATP-competitive protein kinase inhibitor [15]. It has since been shown to bind *P. falciparum* casein kinase 1 (CK1) from blood stage cell lysates [16] and to inhibit the growth a chloroquine-resistant strain of *P. falciparum* (FCR-3) with an IC_50_ of 7.07 ± 0.69 μM [17]. This study describes the effects of kinase inhibitor Purvalanol B application on developmental progression in *P. falciparum* and uses quantitative proteomics to characterize differences in protein expression profiles between wild type and Purvalanol B-treated parasites. Given *P. falciparum* parasite propensity to develop rapid resistance to applied drugs, it is advantageous to determine any changes in protein expression as a result of compound application. By looking at changes in protein compliment, new and sensitive techniques may be able to predict resistance development before it occurs and perhaps develop strategies to stave off the effects of waning drug efficacy.

## Methods

### Maintaining *Plasmodium falciparum* cultures

*Plasmodium falciparum* strain W2 was obtained from Malaria Research and Reference Reagent Resource Center BEI Resources Repository, NIAID, NIH and the strain used was *P. falciparum* W2, MRA-157, deposited by D E Kyle. All experiments with this strain were conducted at biosafety level 2. Parasites were thawed from liquid nitrogen and maintained as previously described with slight modifications [18]. Briefly, parasites were kept in malaria culture medium (MCM) (RPMI 1640 (Gibco), 25 mM HEPES, 23 mM NaHCO_3_, and 25 μg/ml gentamicin sulphate (Sigma-Aldrich), pH 7.4) supplemented with 10% human serum (type AB-) at 5% haematocrit (blood type O^+^) in an atmosphere of 5% CO_2_, 5% O_2_, with a balance of N. Cultures were maintained at 37°C and were split 1:50 when parasitaemia reached 3-5%. Parasite growth synchronization protocol was adapted from methods previously described [19]. Synchronized cultures were split 1:10 and allowed to grow for 48 hours after which blood smears were taken and Giemsa stained (HARLECO) to count parasitaemia levels and estimate life cycle stage. The synchronization procedure was repeated until >90% of parasites were in the same life cycle stage as verified by Giemsa-stained blood smears.

### SYBR® Green I parasite growth assay

In order to determine if Purvalanol B was able to inhibit intra-erythrocytic parasite growth of *P. falciparum* strain W2 and to calculate the 50% inhibitory concentration (IC_50_), a SYBR® Green I parasite growth assay was performed as previously described with minor alterations [20]. Purvalanol B (Tochris Bioscience) was reconstituted in DMSO at 10 mM. IC_50_ determinations were made by diluting 10 mM Purvalanol B stock in a 12 point 1:2 dilution series from a starting final concentration of 200 uM. Each concentration was plated in triplicate and the assay was completed independently three times. Assays were conducted in a 96-well plate format in a total volume of 100 μl (99 μl cMCM with 2% haematocrit at 0.5% parasitaemia, 1 μl DMSO with or without appropriate concentration of Purvalanol B). Negative control wells contained 99 μl of cMCM with 2% haematocrit with no parasites, while positive control wells contained 99 μl of cMCM with 2% haematocrit at 0.5% parasitaemia. In order to control for the effects of DMSO, 1 μl of DMSO was added to all control wells. Plates were kept at 37°C in modular incubators that were gassed every 24 hours at 20 L/min for 5 min with 5% O_2_, 5% CO_2_, and a balance of N. After 72 hours, 100 μl of RBC lysis buffer (20 mM Tris base, 5 mM EDTA, 0.0008% saponin, 0.08% Triton X-100, pH 7.5 with 0.2 μl SYBR® Green I/ml of lysis buffer) was added to each well. Plates were incubated in the dark for 1 hour and then read for fluorescence (excitation 485 nm, emission 535 nm) on a Wallac Victor 2 Multi-label Counter (Perkin Elmer). Final fluorescence readings were analysed with non-linear regression analysis using GraphPad Prism® version 5 software with the sigmoidal dose–response (variable slope) equation. Z factor scores were tabulated for all assays to assess statistical effects size and all scores were found to exceed 0.7.

### Culturing parasites with Purvalanol B

Parasites were maintained and synchronized as described above. At 5% parasitaemia, parasites were resynchronized and placed at 37°C for 24 hours. At 24 hours post invasion, parasitaemia levels and life cycle stage were confirmed with Giemsa-stained blood smears and 3× the IC_50_ concentration (determined by SYBR Green I growth assay to be 29.8 μM) of Purvalanol B in DMSO (no more than 1% of total culture volume) was applied to cultures, which were then gassed and incubated for 12 hours at 37°C. Three treatment cultures and three control cultures were prepared. Control cultures were incubated with DMSO vehicle only for 12 hours. At 36 hours post invasion, parasitaemia and life cycle staged were confirmed with Giemsa-stained blood smears. In order to conduct qPCR analysis, 1 mL of cultures was removed from each culture flask and treated as indicated below. Red blood cells (RBCs) were pelleted and washed 3X in PBS to remove serum proteins. All centrifugation steps took place at 4°C. RBCs were then lysed by adding 20× packed cell volume of 0.05% saponin (Fluka BioChemika) in PBS and allowing the mixture to sit on ice for 10 min. Resulting lysates were then washed 3X in PBS to remove background and once in 10 mM Tris HCl (Sigma-Aldrich) to remove haem-binding proteins before final sedimentation of parasite pellets. Parasites were centrifuged at 8,000 rpm for 10 min and the resulting parasite pellets were stored at −80°C.

### Protein extraction and preparation

Parasite lysates were resuspended in 1 mL of 10 mM Tris HCl supplemented with protease inhibitor cocktail (Sigma-Aldrich) and were flash-frozen 2× to begin protein release. Suspensions were then sonicated on ice 5X for 20 sec (1 sec on and 1 sec off) at 10% amplitude to complete protein release. Samples were centrifuged at 14,000 rpm for 30 min at 4°C to sediment haemozoin and the resulting supernatant was removed for later analysis. Protein concentrations were determined by DC assay (Biorad). For each sample, 60 μg of protein was sent to Colorado State University’s Proteomics and Metabolomics Facility for global proteomics analysis. Each sample underwent in-solution proteolytic digestion as previously described [21]. Briefly, samples were solubilized in 8 M urea, 0.2% Protease Max (Promega, Madison, WI, USA), then reduced with dithiothreitol, alkylated with iodoacetamide, and digested with 1% Protease Max and trypsin at 37°C for 3 h. Samples were dried in a Speed Vac® vacuum centrifuge, desalted using Pierce PepClean C18 spin columns (Pierce, Rockford, IL, USA), dried and resuspended in 30 μL 3% ACN, 0.1% formic acid.

### Global proteomics analysis

First peptides were purified and concentrated using on-line enrichment columns (Thermo Scientific 5 μm, 100 μm ID × 2 cm C18 column). Peptide fragments underwent chromatographic separation on a reverse phase nanospray column (Thermo Scientific EASYnano-LC, 3 μm, 75 μm ID x 100 mm C18 column) using a 90-min linear gradient from 10%-30% buffer B (100% ACN, 0.1% formic acid) at a flow rate of 400 nanolitres/min. Peptides were then eluted directly onto the mass spectrometer (Thermo Scientific Orbitrap Velos Pro) and spectra were collected over a m/z range of 250–2,000 Daltons using a dynamic exclusion limit of 2 MS/MS spectra of a given peptide mass for 30 sec (exclusion duration of 90 sec). Samples (0.5 μg) were analysed in randomized duplicate injections. Compound lists of the resulting spectra were generated using Xcalibur 2.2 software (Thermo Scientific) with a S/N threshold of 1.5 and 1 scan/group.

MS/MS spectra were searched against the Uniprot *P. falciparum* concatenated reverse database (version 03/06/2013) using the Mascot database search engine (version 2.3) and the Sorcerer^TM^SEQUEST® version 3.5. LFDR (local false discovery rate) 1% and was calculated using a Bayesian algorithm to confirm peptide probabilities based on likelihoods calculated using parent mass accuracy. Search parameters were as follows: monoisotopic mass, parent ion mass tolerance of 20 ppm, fragment ion mass tolerance of 0.8 Da, fully tryptic peptides with one missed cleavage, variable modification of oxidation of M and fixed modification of carbamidomethylation of C. Search results for each independently analysed sample were imported into the Scaffold software (Version 4, Proteome Software, Portland, OR, USA). Peptide and protein probability thresholds of 95 and 99%, respectively, were applied and a minimum of two unique peptides was required. Manual validation of MS/MS spectra was performed for all protein identifications above the probability thresholds that were based on only two unique peptides. Criteria for manual validation included the following: 1) minimum of 80% coverage of theoretical y or b ions (at least 5 in order); 2) absence of prominent unassigned peaks greater than 5% of the maximum intensity; and, 3) indicative residue specific fragmentation, such as intense ions N- terminal to proline and immediately C- terminal to aspartate and glutamate (used as additional parameters of confirmation). Scaffold software was used to extract Gene Ontology terms from the UniProt database for each identified *P. falciparum* protein.

### Relative protein quantitation

Spectral counting (SpC) and average total ion current (TIC) relative quantitative analyses were performed and t-tests as well as fold changes were calculated for each method. The cut-off and thresholds used for this analysis were as follows: 1) 99% protein probability; 2) 2 peptide minimum; and, 3) 95% peptide probability. There was a minimum of ten spectral counts (sum) per biological group (i.e. Purvalanol B-treated and control) and a minimum presence (two unique peptides) in two of three biological replicates per biological group. For each biological replicate, a scaling factor was determined by dividing the sum of spectral counts by the average spectral counts across all biological samples. Each protein was divided by the scaling factor of the biological replicate in which it was present. T-tests as well as fold-change analysis were then performed on normalized data in Scaffold. Mean and standard deviation of spectral counts were calculated before and after normalization to assure that normalization had equalled out the mean spectral counts between the biological samples. Proteins with SpC or TIC p-values below 0.05 and fold-change values ≤0.5 were considered down-regulated and proteins with p-values below 0.05 and fold-change value ≥1.5 were considered up-regulated.

### Quantitative real-time RT-PCR

Due to the lack of readily available antibodies for *P. falciparum*, results were confirmed by quantitative real-time RT-PCR, which was performed on total RNA extracted from the same cultures that were analysed by mass spectrometry. Total RNA was extracted from samples with TRIZOL reagent (Invitrogen) and samples were treated with TURBO™ DNase (Ambion) to removed contaminating genomic DNA, according to the manufacturer’s suggested protocols. RNA extracted from each sample was run out on a 1% agarose gel and stained with ethidium bromide to assure that RNA was intact and 260/280 ratios were >2.0 as determined by a NanoDrop 2000 UV–vis Spectrophotometer (Thermo Scientific). Resulting total RNA was used to synthesize first-strand cDNA using SuperScript® III First-Strand Synthesis System for RT-PCR (Invitrogen) according to the manufacturer’s protocol. Gene-specific primers were designed using Primer3 primer tool software version 4.0.0 [22]. The gene for the 18S ribosomal subunit of *P. falciparum* was used as a reference gene with forward primer 5′-GCTCTTTCTTGATTTCTTGGATG-3′ and reverse primer 5′-AGCAGGTTAAGATCTCGTTCG-3′ [23]. For quantitative real-time PCR reactions, 2 μL cDNA, 200 nM of each gene-specific primer, and 5 μL SsoAdvanced™ SYBR® Green Supermix (Bio-Rad) were used in 10 μL total volume reactions. The conditions for PCR were 95°C for 30 sec, 95°C for 5 sec and primer-specific annealing temperature for 15 sec for 40 cycles and a 65-95°C melt curve in 0.5°C increments for 3 min. Primer-specific annealing temperatures were 55°C (GPFC0745c [GenBank:XM_001351204.1] and PFI0370c [GenBank:XM_001351913.1]) and 59°C (PF13_0282 [GenBank:XM_001350214.1] and trxr2 [AF508128.1]) and amplifications were performed in a Biorad CFX384 Touch™ Real Time PCR Detection System running CFX Manager™ Software. Sufficiently efficient primers could not be designed for all genes of interest due to the high adenine/thymine content of the gene-coding sequences and the high degree of non-specific binding observed with unsuitable primer pairs. Results were analysed using the 2^ΔΔCT^ method as previously described [24].

## Results

### Giemsa-stained blood smears

**Figure 1:**
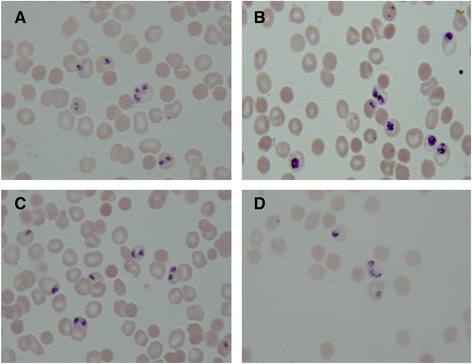
**Control and Purvalanol B-treated parasites before and after inhibitor application. (A)** Representative image of cultured control parasitized RBCs prior to treatment with Purvalanol B; **(B)** representative image of cultured, control, parasitized RBCs after 12-hour treatment period (arrow points to multinucleated cells); **(C)** parasitized RBCs from a treatment flask prior to treatment with Purvalanol B; **(D)** an image of parasitized RBCs 12 hours after Purvalanol B application. All slides were stained with Giemsa.

Giemsa-stained slides analysed before inhibitor treatment indicated all parasite cultures contained approximately 6% parasitized RBCs and were composed of >90% trophozoite parasites with one nucleus (approximately 24 hpi) prior to treatment with Purvalanol B. Student t-test analysis of parasitaemia counts from post-treatment slides indicated no significant change in parasitaemia during the 12-hour Purvalanol B exposure period among culture flasks (p = 0.0066). However, parasite stage differences were observed between the treatment groups and DMSO controls (Figure 1). While control cultures were composed of mostly multinucleated cells, the inhibitor-treated cultures contained predominantly parasites with only one nucleus, thus indicating parasites in the Purvalanol B-treated group were unable to undergo schizogony. A student t-test revealed a significant difference in the number of cells that were able to form multiple nuclei between the control and inhibitor-treated cultures (p = 0.0006).

### Global proteomics analysis

**Figure 2:**
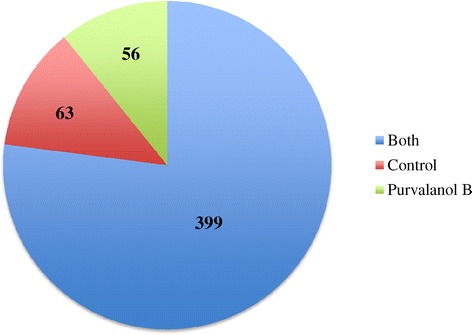
**Number of proteins in control and Purvalanol B-treated samples.** Of the 518 total proteins identified in the *P. falciparum* search, 399 proteins were found in both Purvalanol-B-treated cultures (blue), 63 proteins were found only in control cultures (red) and 56 proteins were found only in Purvalanol B-treated cultures.

Of the six samples analysed, three were control group biological replicates and three were Purvalanol B-treated biological replicates. A total of 52,038 *P. falciparum* spectra and 25,506 human spectra were identified among the six samples. The 52,038 spectra identified in the *P. falciparum* Mascot search corresponded to 518 *P. falciparum* proteins and the 25,506 identified human spectra were assigned to 76 human proteins. Of 518 protein identifications from the *P. falciparum* search, 399 proteins were found both in control and treatment samples, 63 proteins were found in control samples only (Additional file 1) and 56 proteins were identified in Purvalanol B-treated samples only (Additional file 2) (Figure 2).

**Figure 3:**
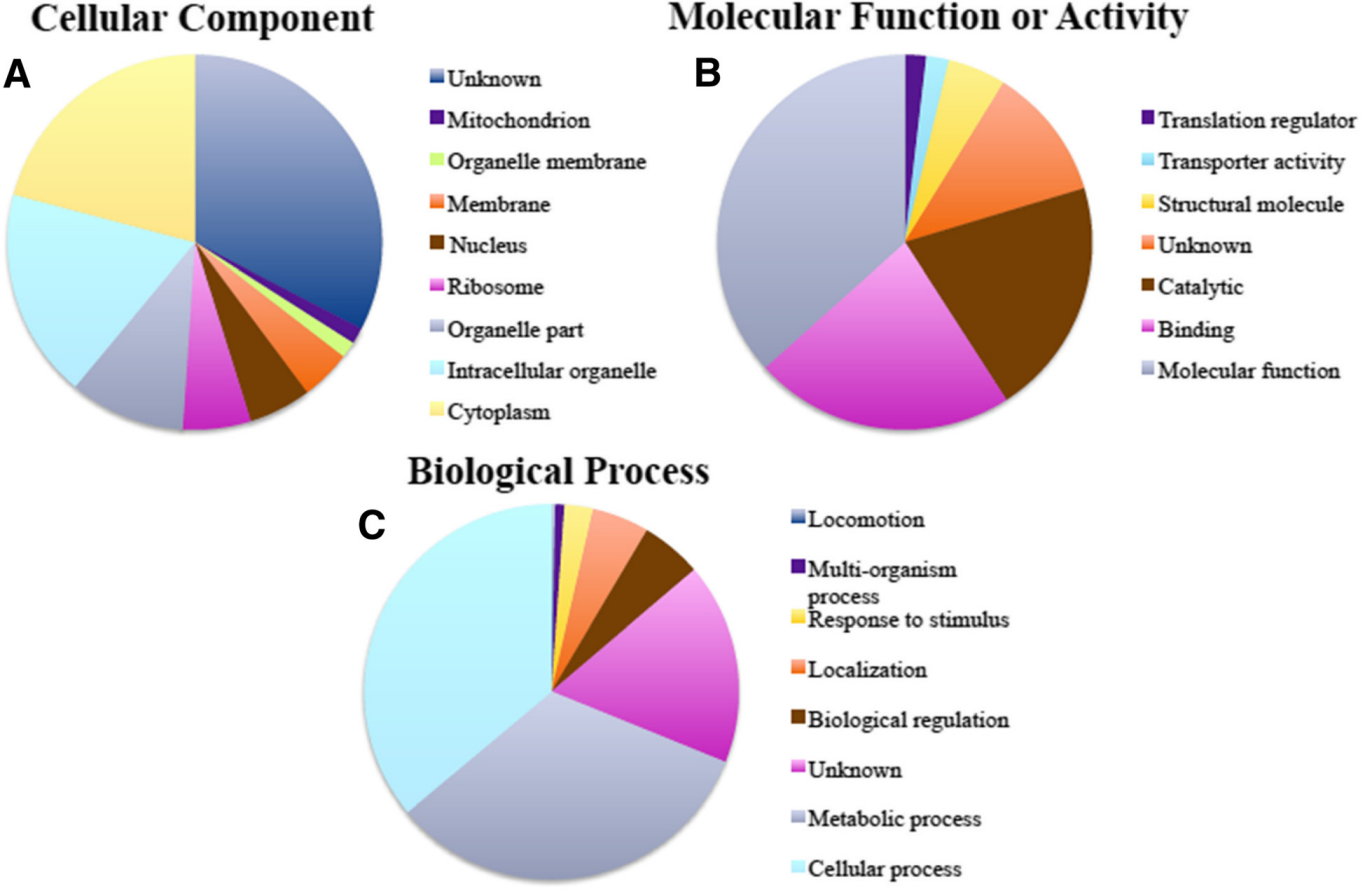
**Gene ontology classification of** ***Plasmodium falciparum*** **proteins. (A)** GO terms describing identified proteins as cellular components. The categories represented in the cellular component category were unknown (dark blue), mitochondrion (purple), organelle membrane (light green), membrane (orange), nucleus (brown), ribosome (fuchsia), organelle part (grey), intracellular organelle (light blue), and cytoplasm (yellow). **(B)** GO terms describing identified proteins in terms of their molecular function or activity. Identified proteins were classified as translation regulator (purple), transporter activity (light blue), structural molecule (yellow), unknown (orange), catalytic (brown), binding (fuchsia), or molecular function (grey). **(C)** GO terms describing identified proteins in terms of their biological function. Identified proteins likely participate in locomotion (dark blue), multi-organism process (purple), response to stimulus (yellow), localization (orange), biological regulation (brown), unknown (fuchsia), metabolic process, and/or cellular process (light blue) within the parasite.

Gene ontology (GO) terms were extracted for all identified *P. falciparum* proteins. The number of proteins categorized into each GO domain and sub-domain were approximately the same between treated and control groups (Additional file 3). No difference in the number of proteins assigned to each GO term existed between treated and control groups. As such, GO terms assigned to proteins from treated and control groups were described together and divided into sub-domains cellular component, molecular function and biological process (Figure 3).

### Relative protein quantitation

SpC and TIC analyses were used to calculate the relative protein quantities of all 518 identified *P. falciparum* proteins (Additional file 4). Both t-test p-values and fold changes were calculated and those proteins that had p-values below 0.05 and fold-change values ≤ 0.5 were considered down-regulated and proteins with p-values below 0.05 and fold-change values ≥ 1.5 were considered up-regulated. Calculation of fold change and t-test p-values indicated ten proteins that were differentially regulated between control and treated groups (Table 1).

**Table 1:** **Differentially regulated proteins between Purvalanol B-treated and control samples**

***Protein name***	***Uniprot accession number***	***P-value***	***Fold change***
Proteasome component C8, putative OS = *Plasmodium falciparum* (isolate 3D7) GN = PFC0745c PE = 4 SV = 1	O77396_PLAF7	0.0016	2.1
Subunit of proteasome activator complex, putative OS = *Plasmodium falciparum* (isolate 3D7) GN = PFI0370c PE = 4 SV = 1	Q8I374_PLAF7	0.015	2
Structure specific recognition protein OS = *Plasmodium falciparum* (isolate 3D7) GN = PF14_0393 PE = 4 SV = 1	Q8IL56_PLAF7	0.027	2.2
Adenylosuccinate synthetase OS = *Plasmodium falciparum* (isolate 3D7) GN = Adss PE = 3 SV = 1	PURA_PLAF7	0.032	1.8
Proteasome subunit alpha type OS = *Plasmodium falciparum* (isolate 3D7) GN = PF13_0282 PE = 3 SV = 1	Q8IDG3_PLAF7	0.038	1.6
Cysteinyl-tRNA synthetase, putative OS = *Plasmodium falciparum* (isolate 3D7) GN = PF10_0149 PE = 3 SV = 2	Q8IJP3_PLAF7	0.04	2.8
Thioredoxin reductase 2 OS = *Plasmodium falciparum* (isolate 3D7) GN = trxr2 PE = 2 SV = 2	TRXR2_PLAF7	0.049	2.9
40S ribosomal protein S6, putative OS = *Plasmodium falciparum* (isolate 3D7) GN = PF13_0228 PE = 4 SV = 1	Q8IDR9_PLAF7	0.037	1.5
Helicase, putative OS = *Plasmodium falciparum* (isolate 3D7) GN = PF14_0437 PE = 3 SV = 2	Q8IL13_PLAF7	0.019	0.3
RNA binding protein, putative OS = *Plasmodium falciparum* (isolate 3D7) GN = PFI1175c PE = 4 SV = 1	Q8I2R8_PLAF7	0.021	0.2

Of the ten differentially regulated proteins, eight were found to be up-regulated. 40S ribosomal protein S6 is a ribosomal protein and cysteinyl-tRNA synthetase an apicoplast- localized protein which are both involved in translation. Adenylosuccinate synthetase is an enzyme that catalyzes the formation of adenylosuccinate from inosine monophosphate and aspartate [25]. Interestingly, *P. falciparum* lacks the necessary synthesis machinery for *de novo* synthesis of purine and thus all purine precursors are salvaged from the host (as reviewed in [26]).

Protein extracts resulting from blood stage *P. falciparum* parasites treated with Purvalanol B and extracts from control parasites administered DMSO vehicle only were analysed with global proteomics analysis and results were relatively quantified. Protein name, Uniprot accession number, p-values and fold changes are reported.

### Quantitative real-time RT-PCR

In order to validate relative protein quantities reported in Table 1, it was necessary to quantify the relative abundances of gene transcripts corresponding to each protein as antibodies for these gene products are not commercially available. Synchronized blood stage cultures of *P. falciparum* at 24 hours post-invasion were treated with 3X the IC_50_ concentration of Purvalanol B and allowed to incubate for a further 12 hours. Total RNA was extracted from 1 mL of infected culture at 5% haematocrit and 6% parasitaemia. Total RNA was reverse transcribed and quantitative real-time reactions were run in triplicate for each gene on each sample. Results were analysed by the 2^ΔΔCT^ method and fold changes and standard deviations were calculated for each amplicon. While all transcript levels could not be assessed due to the difficulty in designing gene-specific primers for some coding sequences, the results from quantitative real-time RT-PCR assays generally agreed with the results obtained from relative protein quantitation (Table 2). Transcript levels of proteasome component C8 [GenBank: XM_001351204.1] were shown to be up-regulated in Purvalanol B-treated cultures compared to control cultures. In addition to proteasome component C8, sub-unit of proteasome activator complex [GenBank:XM_001351913.1], proteasome subunit alpha type [GenBank:XM_001350214.1], and thioredoxin reductase 2 [GenBank:AF508128.1], the protein levels of which were all shown to be up-regulated. Relative protein quantitation calculated a 2.1-, 2-, 1.6- and 2.9-fold increase of these proteins, respectively, in Purvalanol B-treated protein extracts compared to control extracts. Given the similar fold increases seen with the quantitative real-time RT-PCR analyses, there seems to be good agreement between the two methods.

**Table 2:** **Quantitative real-time RT-PCR results from Purvalanol B-treated** ***vs*** **control**

***Description***	***Gene name***	***Forward primer***	***Reverse primer***	***2*** ^***ΔΔCT***^ ***Fold change***	***STD DEV***
Proteasome component C8	PFC0745c	GGTTGTTAAACCAAAGAATG	TGATTTATTCATTAGAAGGAGG	3.2	0.47
Subunit of proteasome activator complex	PFI0370c	AACAAATAAAGATGGGGAAGTATATC	CATGAACAACTTAACCAAGA	2.8	0.37
Proteasome subunit alpha type	PF13_0282	CCAACAGATGCTGAATCG	TTGTAGCAAATGTCCATCAGG	3.6	0.44
Thioredoxin reductase 2	trxr2	CACCTGCTCTTAATAAAGC	GACTTGTTCCATTTATCCACA	5.1	0.84

In order to detect relative transcript levels between Purvalanol B-treated samples and control samples, quantitative real-time PCR was performed. Results were analysed by the 2^ΔΔCT^ method and fold change as well as standard deviations were calculated based on average values from three biological replicates.

## Discussion

This study describes the morphological and proteomic changes in blood stage *P. falciparum* parasites that result from application with kinase inhibitor Purvalanol B. Using a global proteomic approach and relative protein quantitation we have shown that a number of protein are differentially regulated between Purvalanol B-treated and control parasites.

Thioredoxin reductase, which was shown to be up-regulated in drug-treated cultures, is a cytoplasmic protein that is necessary for redox homeostasis. It belongs to the class-I pyridine nucleotide-disulphide oxidoreductase family and has been identified as a potential anti-malarial drug target because its function is essential for parasite survival and a crystal structure is now available for this protein. Thioredoxin reductase in *P. falciparum* functions as an antioxidant that helps relieve the parasite from oxidative stress [27]. Treatment with the inhibitor Purvalanol B may have increased oxidative stress on parasites and resulted in an up-regulation of this protein. Additionally, in other systems, it has been shown that application of proteasome inhibitors in other cells leads to an increased vulnerability to oxidative stress and that heat shock proteins confer a degree of tolerance to oxidative damage [28]. While not expressed at the significance cut-off levels, heat shock proteins 70 [Uniprot:Q8I5F4_PLAF7] (p-value 0.062, fold change 0.09) and heat shock protein 86 [Uniprot:Q8IC05_PLAF7] (p-value 0.099 and fold change 1.2) may have been up-regulated in treatment samples compared to control samples. Interestingly, as previously discussed, the potent anti-malarial compound artemisinin also functions by increasing oxidative stress on the parasite [14,29].

The up-regulated proteins in treatment samples included proteasome component C8 [Uniprot:O77396_PLAF7], which is a cytoplasmic and nuclear protein that is part of the proteasome core complex alpha-sub-unit and is involved in ubiquitin-dependent catabolic processes such as protein degradation. Two additional proteasome proteins were also up-regulated in the Purvalanol B-treated samples compared to controls, sub-unit of proteasome activator complex [Uniprot:Q8I374_PLAF7] and proteasome sub-unit alpha [Uniprot:Q8IDG3_PLAF7]. While some *P. falciparum* proteasome sub-units have been characterized, little is known about these three protein sub-units. However, it has been shown that proteasome inhibitors are able to attenuate intra-erythrocytic *P. falciparum* growth [30]. Further, it has been shown that while the proteasome in other organisms plays a role in diverse cellular processes such as response to stress and metabolic adaptation, the proteasome complex generally has two primary roles: 1) the ubiquitin-dependent proteolytic degradation of aberrantly folded or assembled proteins; and, 2) the control of cell cycle by the temporally precise proteolysis of selected cell cycle modulators, such as cyclins and transcription factors (as reviewed in [31]).

It may be possible that the *P. falciparum* proteasome also plays a role in cell cycle regulation [32]. Application of Purvalanol B to synchronized *P. falciparum* cultures resulted in an inability of the parasite to complete schizogony as evidenced by a significant reduction in the number of parasite cells able to form multiple nuclei when compared to control cultures and an up-regulation of proteasome sub-units in treatment cultures. Purvalanol B application in treatment cultures may have resulted in proteasome inhibition, which in turn lead to up-regulation of proteasome sub-units and the inability to degrade certain cell cycle machinery, such as structure-specific recognition protein [Uniprot:Q8IL56_PLAF7], which is a nuclear protein that is involved in single-stranded DNA binding necessary to stabilize negatively charged DNA when it is unwound.

Interestingly, while the following proteins did not meet both criteria for significance, proliferating cell nuclear antigen [Uniprot:PCNA_PLAF7 (+1)] (p-value 0.063, fold change 1.6), cell division cycle protein 48 homologue [Uniprot:C6KT34_PLAF7] (p-value 0.022, fold change 1.3), and replication factor A-related protein [Uniprot:Q8I3A1_PLAF7] (p-value 0.08, fold change 2.8) may have been up-regulated in treatment samples. Proliferating cell nuclear antigen was previously discussed and is a processivity factor for DNA polymerase. Cell division cycle protein 48 is a homologue of yeast cdc48p and human p97 [33]. The yeast protein was first isolated in yeast mutants and the non-functional cdc48p was found to cause G2/M arrest. Later it was observed that cdc48p also functions during a G1 checkpoint in yeast that is equivalent to the restriction point in mammals and that progression through this checkpoint was mediated by the cdc48p-dependent degradation of cyclin-dependent kinase inhibitor Far1p [34]. The final probable up-regulated cell cycle protein is replication factor A, which is a single-stranded binding protein that, in yeast, is phosphorylated at the G1/S phase transition by cdc2 (CDK1) and dephosphorylated directly prior to mitosis [35]. Additionally, cdc2 is the yeast homologue of PfPK5 in *P. falciparum,* which has been shown to be inhibited by the CDK inhibitor Purvalanol B at 130 nM, a concentration well below the applied concentration in this experiment [36]. PfPK5 activity has also been shown to be necessary for the completion of the intra-erythrocytic life cycle specifically during schizogony [37].

## Conclusions

This study describes the morphological and proteomic changes in *P. falciparum* blood stages parasites resulting from application of kinase inhibitor Purvalanol B. Purvalanol B-treated parasites were unable to form the multinucleated cells indicative of schizogony at 36 hours post invasion. In addition, these data show an up-regulation of proteasome components in Purvalanol B-treated parasites compared to control parasites and may indicate an increase in oxidative stress levels.

## Additional files

Additional file 1:
**Proteins only in** ***Plasmodium falciparum*** **control samples.** The names and accession numbers of all proteins found only in control cultures of *P. falciparum*. Additional file 2:
**Proteins only in** ***Plasmodium falciparum*** **treatment samples.** The names and accession numbers of all proteins found only in Purvalanol B-treated cultures of *P. falciparum*. Additional file 3:
**Gene ontology terms for control** ***vs*** **Purvalanol B-treated proteins.** Table compares control protein samples to Purvalanol B-treated protein samples in terms of percentage of displayed proteins that fall into each of the 3 GO term categories and their associated sub-categories. Additional file 4:
**Quantitative proteomics data.** File includes multiple spreadsheets containing exclusive unique peptide counts, total spectral counts, quantitative values for normalized spectra, and quantitative values for normalized average total ion current for each quantified protein.

## References

[1] WHO (2012). World malaria report.

[2] Ndong IC, van Reenen M, Boakye DA, Mbacham WF, Grobler AF (2014). Trends in malaria admissions at the Mbakong Health Centre of the North West Region of Cameroon: a retrospective study. Malar J.

[3] Imwong M, Dondorp AM, Nosten F, Yi P, Mungthin M, Hanchana S (2010). Exploring the contribution of candidate genes to artemisinin resistance in *Plasmodium falciparum*. Antimicrob Agents Chemother.

[4] Bosman P, Stassijns J, Nackers F, Canier L, Kim N, Khim S (2014). Plasmodium prevalence and artemisinin-resistant falciparum malaria in Preah Vihear Province, Cambodia: a cross-sectional population-based study. Malar J.

[5] Thriemer K, Van Hong H, Rosanas-Urgell A, Phuc BZ, Ha DM, Pockele E (2014). Delayed parasite clearance after treatment with dihydroartemisinin-piperaquine in *Plasmodium falciparum* malaria patients in Central Vietnam. Antimicrob Agents Chemother.

[6] Mita T, Jombart T (2015). Patterns and dynamics of genetic diversity in *Plasmodium falciparum*: what past human migrations tell us about malaria. Parasitol Int.

[7] Florens L, Washburn MP, Raine JD, Anthony RM, Grainger M, Haynes JD (2002). A proteomic view of the *Plasmodium falciparum* life cycle. Nature.

[8] Nirmalan N, Sims PF, Hyde JE (2004). Quantitative proteomics of the human malaria parasite *Plasmodium falciparum* and its application to studies of development and inhibition. Mol Microbiol.

[9] Pease BN, Huttlin EL, Jedrychowski MP, Talevich E, Harmon J, Dillman T (2013). Global analysis of protein expression and phosphorylation of three stages of *Plasmodium falciparum* intraerythrocytic development. J Proteome Res.

[10] Cooper RA, Carucci DJ (2004). Proteomic approaches to studying drug targets and resistance in Plasmodium. Curr Drug Targets Infect Disord.

[11] Koncarevic S, Bogumil R, Becker K (2007). SELDI-TOF-MS analysis of chloroquine resistant and sensitive *Plasmodium falciparum* stratins. Proteomics.

[12] Prieto JH, Koncarevic S, Park SK, Yates JR, Becker K (2008). Large-scale differential proteome analysis in *Plasmodium falciparum* under drug treatment. PLoS One.

[13] Briolant S, Almeras L, Belghazi M, Boucomont-Chapeaublanc E, Wurtz N, Fontaine A (2010). *Plasmodium falciparum* proteome changes in response to doxycycline treatment. Malar J.

[14] Makanga M, Bray PG, Horrocks P, Ward SA (2005). Towards a proteomic definition of CoArtem action in *Plasmodium falciparum* malaria. Proteomics.

[15] Gray N, Wodicka LM, Thunnissen AWH, Norman TC, Kwon S, Espinoza FH (1998). Exploiting chemical libraries, structure, and genomics in the search for kinase inhibitors. Science.

[16] Knockaert M, Gray N, Damiens E, Chang YT, Grellier P, Grant K (2000). Intracellular targets of cyclin-dependent kinase inhibitors: identification by affinity chromatography using immobilised inhibitors. Chem Biol.

[17] Harmse L, Van Zyl R, Gray N, Schultz P, Leclerc S, Meijer L (2001). Structure-activity relationships and inhibitory effects of various purine derivatives on the in vitro growth of *Plasmodium falciparum*. Biochem Pharmacol.

[18] Trager W, Jensen JB (1976). Human malaria parasites in continuous culture. Science.

[19] Lambros C, Vanderberg JP (1979). Synchronization of *Plasmodium falciparum* erythrocytic stages in culture. J Parasitol.

[20] Smilkstein M, Sriwilaijaroen N, Kelly JX, Wilairat P, Riscoe M (2004). Simple and inexpensive fluorescence-based technique for high-throughput antimalarial drug screening. Antimicrob Agents Chemother.

[21] Freund DM, Prenni JE, Curthoys NP (2013). Response of the mitochondiral proteome of rat renal proximal convoluted tubules to chronic metabolic acidosis. Am J Physiol Renal Physiol.

[22] Primer3. [http://primer3.ut.ee]

[23] Kamau E, Tolbert LS, Kortepeter L, Pratt M, Nyakoe N, Muringo L (2011). Development of a hightly sensitive genus-specific quantitative reverse transcriptase real-time PCR assay for detection and quantitation of Plasmodium by amplifying RNA and DNA of the 18S rRNA genes. J Clin Microbiol.

[24] Livak KJ, Schmittgen TD (2001). Analysis of relative gene expression data using real-time quantitative RCR and the 2(−delta delta C(T)) method. Methods.

[25] Benson CE, Love SH, Remy CN (1970). Inhibition of de novo purine biosynthesis and interconversion by 6-methylpurine in *Escherichia coli*. J Bacteriol.

[26] Downie MJ, Kirk K, Mamoun CB (2008). Purine salvage pathways in the intraerythrocytic malaria parasite *Plasmodium falciparum*. Eukaryot Cell.

[27] Kehr S, Sturm N, Rahlfs S, Przyborski JM, Becker K (2012). Compartmentation of redox metabolism in malaria parasites. PLoS Pathog.

[28] Ding Q, Keller JN (2001). Proteasome inhibition in oxidative stress neurotoxicity: implications for heat shock proteins. J Neurochem.

[29] Antione T, Fisher N, Amewu R, O’Neill PM, Ward SA, Biagini GA (2014). Rapid kill of malaria parasites by artemisinin and semi-synthetic endoperoxides involves ROS-dependent depolarization of the membrane potential. J Antimicrob Chemother.

[30] Gantt SM, Myung JM, Briones MRS, Li WD, Corey EJ, Omura S (1998). Proteasome inhibitors block development of Plasmodium spp. Antimicrob Agents Chemother.

[31] Ichihara A, Tanaka K (1995). Roles of proteasomes in cell growth. Mol Biol Rep.

[32] Paugam A, Bulteau A, Dupouy-Carnet J, Creuzet C, Friguet B (2003). Characterization and role of protozoan parasite proteasomes. Trends Parasitol.

[33] Frohlich KU, Fries HW, Rudinger M, Erdmann R, Botstein D, Mecke D (1991). Yeast cell cycle protein CDC48p shows full-length homology to the mammalian protein VCP and is a member of a protein family involved in secretion, peroxisome formation, and gene expression. J Cell Biol.

[34] Fu X, Ng C, Feng D, Liang C (2003). Cdc48p is required for the cell cycle commitment point at Start via degradation of the G1-CDK inhibitor Far1p. J Cell Biol.

[35] Dutta A, Bell SP (1997). Initiation of DNA replication in eukaryotic cells. Annu Rev Cell Dev Biol.

[36] Holton S, Merckx A, Burgess D, Doerig C, Noble M, Endicott J (2003). Structures of P. falciparum PfPK5 test the CDK regulation paradigm and suggest mechanisms of small molecule inhibition. Structure.

[37] Solyakov L, Halbert J, Alam MM, Semblat JP, Reininger L, Bottrill AR (2011). Global kinomic and phospho-proteomic analyses of the human malaria parasite *Plasmodium falciparum*. Nat Commun.

