# Supplementary material for: Effects of cyclin-dependent kinase inhibitor Purvalanol B application on protein expression and developmental progression in intra-erythrocytic Plasmodium falciparum parasites
Source: Malar J. 2015 Apr 8;14:147. doi: 10.1186/s12936-015-0655-x (PMC4403934; doi:10.1186/s12936-015-0655-x)
Supplement: Additional file 1: — Proteins only in Plasmodium falciparum control samples. The names and accession numbers of all proteins found only in control cultures of P. falciparum. [file 12936_2015_655_MOESM1_ESM.pdf]

Proteins in Only P. falciparum *Control Samples*. Column one provides the protein name and column two the accession number of each protein. Any identifications made from the reverse concatenated database are not represented.

Protein	*Accession Number*
Putative uncharacterized protein OS=Plasmodium falciparum (isolate 3D7) GN=MAL8P1.103 PE=4 SV=1	C0H4U0_PLAF7
Conserved Plasmodium protein OS=Plasmodium falciparum (isolate 3D7) GN=PFL2120w PE=4 SV=1	Q8I4Y9_PLAF7
Putative uncharacterized protein OS=Plasmodium falciparum (isolate 3D7) GN=PFE1465w PE=4 SV=1	C0H4G7_PLAF7
DNA repair protein, putative OS=Plasmodium falciparum (isolate 3D7) GN=PFE0270c PE=3 SV=1	Q8I447_PLAF7
Histidine--tRNA ligase, putative OS=Plasmodium falciparum (isolate 3D7) GN=PF14_0428 PE=4 SV=1	Q8IL22_PLAF7
40S ribosomal protein S28e, putative OS=Plasmodium falciparum (isolate 3D7) GN=PF14_0585 PE=4 SV=1	Q8IKL9_PLAF7
10b antigen, putative OS=Plasmodium falciparum (isolate 3D7) GN=PF10_0213 PE=4 SV=1	Q8IJI4_PLAF7
Ubiquitination-mediated degradation component, putative OS=Plasmodium falciparum (isolate 3D7) GN=PF08_0020 PE=4 SV=1	C0H4Y0_PLAF7
Putative uncharacterized protein OS=Plasmodium falciparum (isolate 3D7) GN=PF07_0016 PE=4 SV=1	Q8IC27_PLAF7
Vacuolar ATP synthase subunit E, putative OS=Plasmodium falciparum (isolate 3D7) GN=PFI1670c PE=3 SV=2	Q8I2H3_PLAF7
V-type ATPase, subunit C, putative OS=Plasmodium falciparum (isolate 3D7) GN=PFA_0300c PE=4 SV=1	Q8I280_PLAF7
Arginyl-tRNA synthetase, putative OS=Plasmodium falciparum (isolate 3D7) GN=PFL0900c PE=3 SV=1	Q8I5M2_PLAF7
Conserved Plasmodium protein OS=Plasmodium falciparum (isolate 3D7) GN=PF11_0332 PE=4 SV=1	Q8II42_PLAF7
Putative uncharacterized protein OS=Plasmodium falciparum (isolate 3D7) GN=PFE0990w PE=4 SV=1	C0H4F1_PLAF7
Proteasome, putative OS=Plasmodium falciparum (isolate 3D7) GN=PFI1545c PE=4 SV=1	Q8I0U7_PLAF7
DNA-directed RNA polymerase OS=Plasmodium falciparum (isolate 3D7) GN=PFC0805w PE=3 SV=1	O77375_PLAF7
P-type calcium transporting ATPase OS=Plasmodium falciparum GN=serca PE=3 SV=1	E1CC54_PLAFA
60S ribosomal protein L23a, putative OS=Plasmodium falciparum (isolate 3D7) GN=PF13_0132 PE=3 SV=1	Q8IE82_PLAF7
Coatomer alpha subunit, putative OS=Plasmodium falciparum (isolate 3D7) GN=PFF0330w PE=4 SV=1	C6KSR5_PLAF7
Pfmdr2 protein OS=Plasmodium falciparum GN=pfmdr2 PE=3 SV=1	Q25693_PLAFA
60S ribosomal protein L14, putative OS=Plasmodium falciparum (isolate 3D7) GN=PF14_0296 PE=4 SV=1	Q8ILE8_PLAF7

Protein	*Accession Number*
Putative uncharacterized protein OS=Plasmodium falciparum (isolate 3D7) GN=MAL8P1.103 PE=4 SV=1	C0H4U0_PLAF7
Conserved Plasmodium protein OS=Plasmodium falciparum (isolate 3D7) GN=PFL2120w PE=4 SV=1	Q8I4Y9_PLAF7
Putative uncharacterized protein OS=Plasmodium falciparum (isolate 3D7) GN=PFE1465w PE=4 SV=1	C0H4G7_PLAF7
DNA repair protein, putative OS=Plasmodium falciparum (isolate 3D7) GN=PFE0270c PE=3 SV=1	Q8I447_PLAF7
Histidine--tRNA ligase, putative OS=Plasmodium falciparum (isolate 3D7) GN=PF14_0428 PE=4 SV=1	Q8IL22_PLAF7
40S ribosomal protein S28e, putative OS=Plasmodium falciparum (isolate 3D7) GN=PF14_0585 PE=4 SV=1	Q8IKL9_PLAF7
10b antigen, putative OS=Plasmodium falciparum (isolate 3D7) GN=PF10_0213 PE=4 SV=1	Q8IJI4_PLAF7
Ubiquitination-mediated degradation component, putative OS=Plasmodium falciparum (isolate 3D7) GN=PF08_0020 PE=4 SV=1	C0H4Y0_PLAF7
Putative uncharacterized protein OS=Plasmodium falciparum (isolate 3D7) GN=PF07_0016 PE=4 SV=1	Q8IC27_PLAF7
Vacuolar ATP synthase subunit E, putative OS=Plasmodium falciparum (isolate 3D7) GN=PFI1670c PE=3 SV=2	Q8I2H3_PLAF7
V-type ATPase, subunit C, putative OS=Plasmodium falciparum (isolate 3D7) GN=PFA_0300c PE=4 SV=1	Q8I280_PLAF7
Arginyl-tRNA synthetase, putative OS=Plasmodium falciparum (isolate 3D7) GN=PFL0900c PE=3 SV=1	Q8I5M2_PLAF7
Conserved Plasmodium protein OS=Plasmodium falciparum (isolate 3D7) GN=PF11_0332 PE=4 SV=1	Q8II42_PLAF7
Putative uncharacterized protein OS=Plasmodium falciparum (isolate 3D7) GN=PFE0990w PE=4 SV=1	C0H4F1_PLAF7
Proteasome, putative OS=Plasmodium falciparum (isolate 3D7) GN=PFI1545c PE=4 SV=1	Q8I0U7_PLAF7
DNA-directed RNA polymerase OS=Plasmodium falciparum (isolate 3D7) GN=PFC0805w PE=3 SV=1	O77375_PLAF7
P-type calcium transporting ATPase OS=Plasmodium falciparum GN=serca PE=3 SV=1	E1CC54_PLAFA
60S ribosomal protein L23a, putative OS=Plasmodium falciparum (isolate 3D7) GN=PF13_0132 PE=3 SV=1	Q8IE82_PLAF7
Coatomer alpha subunit, putative OS=Plasmodium falciparum (isolate 3D7) GN=PFF0330w PE=4 SV=1	C6KSR5_PLAF7
Pfmdr2 protein OS=Plasmodium falciparum GN=pfmdr2 PE=3 SV=1	Q25693_PLAFA
60S ribosomal protein L14, putative OS=Plasmodium falciparum (isolate 3D7) GN=PF14_0296 PE=4 SV=1	Q8ILE8_PLAF7
