# Supplementary material for: Effects of cyclin-dependent kinase inhibitor Purvalanol B application on protein expression and developmental progression in intra-erythrocytic Plasmodium falciparum parasites
Source: Malar J. 2015 Apr 8;14:147. doi: 10.1186/s12936-015-0655-x (PMC4403934; doi:10.1186/s12936-015-0655-x)
Supplement: Additional file 2: — Proteins only in Plasmodium falciparum treatment samples. The names and accession numbers of all proteins found only in Purvalanol B-treated cultures of P. falciparum. [file 12936_2015_655_MOESM2_ESM.pdf]

Proteins Only in P. falciparum *Treatment Samples*. Column one provides the protein name and column two the accession number for each protein. Any identifications made from the reverse concatenated database are not represented.

Protein	*Accession Number*
Putative uncharacterized protein OS=Plasmodium falciparum (isolate 3D7) GN=MAL8P1.103 PE=4 SV=1	C0H4U0_PLAF7
Conserved Plasmodium protein OS=Plasmodium falciparum (isolate 3D7) GN=PFL2120w PE=4 SV=1	Q8I4Y9_PLAF7
Putative uncharacterized protein OS=Plasmodium falciparum (isolate 3D7) GN=PFE1465w PE=4 SV=1	C0H4G7_PLAF7
DNA repair protein, putative OS=Plasmodium falciparum (isolate 3D7) GN=PFE0270c PE=3 SV=1	Q8I447_PLAF7
Histidine--tRNA ligase, putative OS=Plasmodium falciparum (isolate 3D7) GN=PF14_0428 PE=4 SV=1	Q8IL22_PLAF7
40S ribosomal protein S28e, putative OS=Plasmodium falciparum (isolate 3D7) GN=PF14_0585 PE=4 SV=1	Q8IKL9_PLAF7
10b antigen, putative OS=Plasmodium falciparum (isolate 3D7) GN=PF10_0213 PE=4 SV=1	Q8IJI4_PLAF7
Ubiquitination-mediated degradation component, putative OS=Plasmodium falciparum (isolate 3D7) GN=PF08_0020 PE=4 SV=1	C0H4Y0_PLAF7
Putative uncharacterized protein OS=Plasmodium falciparum (isolate 3D7) GN=PF07_0016 PE=4 SV=1	Q8IC27_PLAF7
Vacuolar ATP synthase subunit E, putative OS=Plasmodium falciparum (isolate 3D7) GN=PFI1670c PE=3 SV=2	Q8I2H3_PLAF7
V-type ATPase, subunit C, putative OS=Plasmodium falciparum (isolate 3D7) GN=PFA_0300c PE=4 SV=1	Q8I280_PLAF7
Arginyl-tRNA synthetase, putative OS=Plasmodium falciparum (isolate 3D7) GN=PFL0900c PE=3 SV=1	Q8I5M2_PLAF7
Conserved Plasmodium protein OS=Plasmodium falciparum (isolate 3D7) GN=PF11_0332 PE=4 SV=1	Q8II42_PLAF7
Putative uncharacterized protein OS=Plasmodium falciparum (isolate 3D7) GN=PFE0990w PE=4 SV=1	C0H4F1_PLAF7
Proteasome, putative OS=Plasmodium falciparum (isolate 3D7) GN=PFI1545c PE=4 SV=1	Q8I0U7_PLAF7

Protein	*Accession Number*
DNA-directed RNA polymerase OS=Plasmodium falciparum (isolate 3D7) GN=PFC0805w PE=3 SV=1	O77375_PLAF7
P-type calcium transporting ATPase OS=Plasmodium falciparum GN=serca PE=3 SV=1	E1CC54_PLAFA
60S ribosomal protein L23a, putative OS=Plasmodium falciparum (isolate 3D7) GN=PF13_0132 PE=3 SV=1	Q8IE82_PLAF7
Coatomer alpha subunit, putative OS=Plasmodium falciparum (isolate 3D7) GN=PFF0330w PE=4 SV=1	C6KSR5_PLAF7
Pfmdr2 protein OS=Plasmodium falciparum GN=pfmdr2 PE=3 SV=1	Q25693_PLAFA
60S ribosomal protein L14, putative OS=Plasmodium falciparum (isolate 3D7) GN=PF14_0296 PE=4 SV=1	Q8ILE8_PLAF7
Heat shock protein 90, putative OS=Plasmodium falciparum (isolate 3D7) GN=PF11_0188 PE=3 SV=1	Q8III6_PLAF7
ORF 2 protein (Fragment) OS=Plasmodium falciparum GN=ORF 2 PE=2 SV=1	Q02602_PLAFA
Ring-exported protein 1 OS=Plasmodium falciparum (isolate 3D7) GN=REX1 PE=4 SV=1	Q8I2G1_PLAF7
40S ribosomal protein S24 OS=Plasmodium falciparum (isolate 3D7) GN=PFE0975c PE=3 SV=1	Q8I3R6_PLAF7
Glutamate--cysteine ligase (Gamma-glutamylcysteine synthetase) OS=Plasmodium falciparum GN=GCS PE=2 SV=1	Q9TY17_PLAFA
Activator of Hsp90 ATPase homolog 1-like protein, putative OS=Plasmodium falciparum (isolate 3D7) GN=PFC0360w PE=4 SV=2	O97256_PLAF7
Ubiquitin conjugating enzyme E2, putative OS=Plasmodium falciparum (isolate 3D7) GN=PFL0190w PE=3 SV=1	Q8I607_PLAF7
Coatamer protein, beta subunit, putative OS=Plasmodium falciparum (isolate 3D7) GN=PF14_0277 PE=4 SV=2	Q8ILG6_PLAF7
Putative uncharacterized protein OS=Plasmodium falciparum (isolate 3D7) GN=MAL8P1.62 PE=4 SV=1	Q8IB31_PLAF7
60S ribosomal protein L36 OS=Plasmodium falciparum (isolate 3D7) GN=PF11_0106 PE=3 SV=2	Q8I713_PLAF7

Protein	*Accession Number*
Serine rich protein homologue OS=Plasmodium falciparum PE=3 SV=1	Q26015_PLAFA
6-cysteine protein, putative OS=Plasmodium falciparum (isolate 3D7) GN=Pf41 PE=4 SV=1	Q8I1Y0_PLAF7
DNA replication licensing factor mcm7 homologue, putative OS=Plasmodium falciparum (isolate 3D7) GN=PF07_0023 PE=3 SV=1	Q8IC16_PLAF7
Rab2, GTPase OS=Plasmodium falciparum (isolate 3D7) GN=Rab2 PE=3 SV=1	Q8I5A9_PLAF7
Cytidine triphosphate synthetase OS=Plasmodium falciparum (isolate 3D7) GN=PF14_0100 PE=4 SV=1	Q8ILZ3_PLAF7
Ubiquitin carboxyl-terminal hydrolase OS=Plasmodium falciparum (isolate 3D7) GN=PFE1355c PE=3 SV=1	Q8I3J3_PLAF7
Glycogen synthase kinase 3 OS=Plasmodium falciparum (isolate 3D7) GN=PfGSK-3 PE=4 SV=2	O77344_PLAF7
Acetyl-CoA synthetase OS=Plasmodium falciparum (isolate 3D7) GN=PFF1350c PE=4 SV=1	C6KTB4_PLAF7
DNAJ protein, putative OS=Plasmodium falciparum (isolate 3D7) GN=PF08_0032 PE=4 SV=1	Q8IB72_PLAF7
DNA topoisomerase 2 OS=Plasmodium falciparum (isolate 3D7) GN=PF14_0316 PE=3 SV=1	Q8ILC8_PLAF7
Conserved Plasmodium protein OS=Plasmodium falciparum (isolate 3D7) GN=PFL0895c PE=4 SV=1	Q8I5M3_PLAF7
Spermidine synthase OS=Plasmodium falciparum (isolate 3D7) GN=PF11_0301 PE=1 SV=1	Q8II73_PLAF7
Putative uncharacterized protein OS=Plasmodium falciparum (isolate 3D7) GN=PF08_0137 PE=4 SV=1	Q8IAK9_PLAF7
PfSec23 protein OS=Plasmodium falciparum (isolate 3D7) GN=Pfsec23 PE=2 SV=1	Q8IB60_PLAF7
Replication factor C subunit 1 OS=Plasmodium falciparum GN=rfc1 PE=4 SV=1	Q9GQW6_PLAFA
60S ribosomal protein L8, putative OS=Plasmodium falciparum (isolate 3D7) GN=PFE0845c PE=4 SV=1	Q8I3T9_PLAF7

Protein	*Accession Number*
Ubiquitin carboxyl-terminal hydrolase a, putative OS=Plasmodium falciparum (isolate 3D7) GN=PFD0680c PE=4 SV=1	Q8I1U8_PLAF7
Orotate phosphoribosyltransferase OS=Plasmodium falciparum GN=oprt PE=2 SV=1	Q8N0R1_PLAFA
Calmodulin OS=Plasmodium falciparum (isolate 3D7) GN=PF14_0323 PE=3 SV=2	CALM_PLAF7
Haloacid dehalogenase-like hydrolase, putative OS=Plasmodium falciparum (isolate 3D7) GN=PF11_0190 PE=4 SV=1	Q8III4_PLAF7
Thymidylate kinase, putative OS=Plasmodium falciparum (isolate 3D7) GN=TMK PE=1 SV=1	Q8I4S1_PLAF7
Myosin-A OS=Plasmodium falciparum (isolate FCBR / Columbia) PE=2 SV=1	MYOA_PLAFB
Probable DNA-directed RNA polymerase II subunit RPB11 OS=Plasmodium falciparum (isolate 3D7) GN=PF13_0023 PE=3 SV=1	RPB11_PLAF7
Zinc finger protein, putative OS=Plasmodium falciparum (isolate 3D7) GN=PF13_0313 PE=4 SV=1	Q8IDC0_PLAF7
60S ribosomal protein L28, putative OS=Plasmodium falciparum (isolate 3D7) GN=PF11_0437 PE=4 SV=1	Q8IHU0_PLAF7
