# Supplementary figures and images for: Effects of cyclin-dependent kinase inhibitor Purvalanol B application on protein expression and developmental progression in intra-erythrocytic Plasmodium falciparum parasites
Source: Malar J. 2015 Apr 8;14:147. doi: 10.1186/s12936-015-0655-x (PMC4403934; doi:10.1186/s12936-015-0655-x)

## Cellular Component

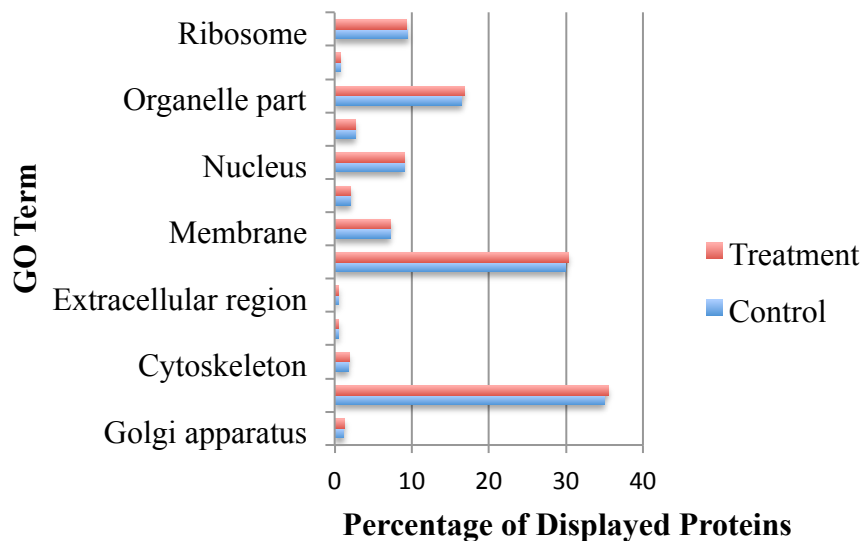

## Molecular Function or Activity

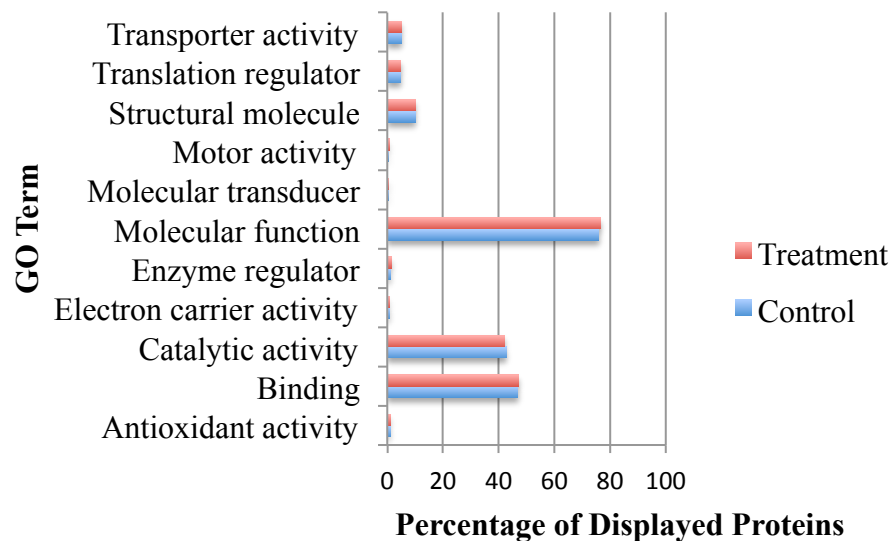

## Biological Process

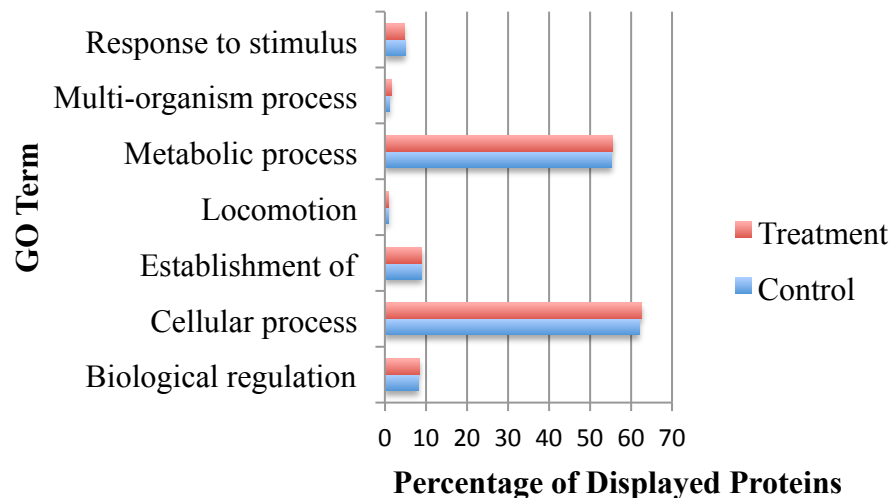

Supplement: Additional file 3: — Gene ontology terms for control vs Purvalanol B-treated proteins. Table compares control protein samples to Purvalanol B-treated protein samples in terms of percentage of displayed proteins that fall into each of the 3 GO term categories and their associated sub-categories. [file 12936_2015_655_MOESM3_ESM.pdf]
